# Development of a risk score for intramyocardial hemorrhage in elderly STEMI patients after primary PCI: a retrospective cohort study with propensity score matching analysis

**DOI:** 10.3389/fcvm.2026.1786172

**Published:** 2026-07-03

**Authors:** Rui Wang, Cuijun Hao, Jiaqi Liu, Zhanshuai Zhang, Shaoqiang Qin

**Affiliations:** 1 Department of Cardiology, The First Affiliated Hospital of Hebei North University, Zhangjiakou, China; 2 Graduate School, Hebei North University, Zhangjiakou, China

**Keywords:** cardiac magnetic resonance, elderly, intramyocardial hemorrhage, percutaneous coronary intervention, risk factors, ST-segment elevation myocardial infarction

## Abstract

**Objective:**

This study aimed to identify independent risk factors for intramyocardial hemorrhage (IMH) and develop a clinical risk score for predicting IMH in elderly patients with ST-segment elevation myocardial infarction (STEMI) undergoing primary percutaneous coronary intervention (PCI).

**Methods:**

We conducted a single-center retrospective observational study of 232 consecutive elderly patients (≥60 years) with STEMI who underwent primary PCI between August 2020 and August 2024 and completed cardiac magnetic resonance (CMR) imaging 3–7 days post-procedure. Patients were classified into IMH (*n* = 77) and non-IMH (*n* = 155) groups based on CMR findings. Clinical, laboratory, angiographic, and procedural data were collected. Univariate and multivariate logistic regression analyses were performed to identify independent predictors. A risk score (IMH-RS) was developed and validated internally.

**Results:**

The incidence of IMH was 33.2% (77/232). Multivariate logistic regression identified the following independent predictors: renal insufficiency (OR = 2.95, 95% CI: 1.41–6.18, *P* = 0.004), left anterior descending artery involvement (OR = 2.01, 95% CI: 1.02–3.97, *P* = 0.044), door-to-balloon time >45 min (OR = 2.55, 95% CI: 1.36–4.78, *P* = 0.003), triple-vessel disease (OR = 6.12, 95% CI: 2.51–14.88, *P* < 0.001), elevated B-type natriuretic peptide (per 100 pg/L increase: OR = 1.25, 95% CI: 1.05–1.49, *P* = 0.012), and reduced left ventricular ejection fraction (per 1% increase: OR = 0.94, 95% CI: 0.91–0.97, *P* < 0.001). An integer-based risk score (IMH-RS) ranging from 0 to 10 was developed, demonstrating good discrimination (AUC = 0.82, 95% CI: 0.77–0.87) and effectively stratifying patients into low- (12.5% IMH), intermediate- (38.2%), and high-risk (68.9%) groups (*P* < 0.001).

**Conclusion:**

IMH is common in elderly STEMI patients after primary PCI and is independently associated with renal dysfunction, extensive coronary disease, delayed reperfusion, and markers of myocardial injury. The IMH-RS score, derived from a comprehensive integration of patient comorbidities, coronary anatomy, procedural timing, and myocardial injury severity, represents a novel and practical bedside tool for early risk stratification. This integrative approach provides a more holistic risk assessment than individual predictors alone. The score may guide targeted management strategies to mitigate microvascular injury.

## 1 Introduction

Acute ST-segment elevation myocardial infarction (STEMI) is one of the most critical forms of cardiovascular disease, characterized by high mortality. Previous studies have shown that the in-hospital mortality of STEMI ranges from 4% to 12%, while the overall 1-year mortality approaches 10% (1, 2). Primary percutaneous coronary intervention (PCI) is the preferred reperfusion strategy for STEMI, as it effectively restores patency of the infarct-related artery and significantly improves clinical outcomes (3). However, despite successful epicardial reperfusion, approximately half of patients still experience inadequate myocardial-level perfusion, known as the “no-reflow” phenomenon, primarily driven by microvascular obstruction (MVO) (4). MVO can further progress to intramyocardial hemorrhage (IMH), a severe manifestation of microvascular injury. IMH exacerbates inflammation and adverse myocardial remodeling, thereby worsening prognosis (5). In particular, IMH is strongly associated with heart failure, malignant arrhythmias, and cardiac death (1, 6).

Although PCI has been widely adopted and its efficacy is well established, microvascular injury remains a key factor limiting optimal clinical benefit in a subset of patients. Cardiac magnetic resonance (CMR) has emerged as the reference standard for visualizing myocardial damage after STEMI, including the detection of IMH (7, 8). However, limited studies have focused specifically on the independent risk factors for IMH after PCI in elderly STEMI patients, particularly within the Chinese population. Existing literature mainly emphasizes imaging or biochemical markers, whereas comprehensive evaluations integrating clinical and procedural characteristics are scarce (6, 9).

Therefore, this study aimed to systematically investigate the independent risk factors for IMH after PCI in elderly STEMI patients through a retrospective analysis, with the goal of constructing a risk-prediction framework to facilitate early identification of high-risk patients and inform future development of targeted microvascular protection strategies.

## 2 Materials and methods

### 2.1 Study design and setting

This single-center, retrospective, observational study was conducted at the Department of Cardiology, The First Affiliated Hospital of Hebei North University. The study was designed to investigate the incidence and independent risk factors of IMH in elderly patients (≥60 years) with acute STEMI who underwent primary PCI. The study period spanned from August 1, 2020, to August 31, 2024. All patients were managed according to the institutional STEMI protocol, which emphasizes early revascularization and standardized post-procedural care. The study was conducted in accordance with the Declaration of Helsinki. Given the retrospective design, the requirement for written informed consent was waived. This study was approved by the ethic committee of The First Affiliated Hospital of Hebei North University.

### 2.2 Patient selection and inclusion/exclusion criteria

We retrospectively screened all consecutive patients aged ≥60 years who were admitted with a diagnosis of STEMI and underwent primary PCI during the study period. The diagnosis of STEMI was based on the 2020 European Society of Cardiology (ESC) guidelines, which require typical chest pain lasting >20 min, persistent ST-segment elevation ≥1 mm in two contiguous leads, and elevated cardiac troponin levels. Patients were included if they: (1) underwent successful primary PCI within 12 h of symptom onset, defined as restoration of Thrombolysis in Myocardial Infarction (TIMI) grade 3 flow with residual stenosis <10%; (2) completed a comprehensive CMR examination 3–7 days after PCI; and (3) had complete clinical and angiographic data available. Exclusion criteria were: (1) prior myocardial infarction or coronary artery bypass grafting; (2) severe renal impairment (baseline serum creatinine >200 *μ*mol/L or end-stage renal disease requiring dialysis); (3) active malignancy, hematologic disorders, or autoimmune diseases; (4) history of significant valvular heart disease, hypertrophic cardiomyopathy, or heart failure with reduced ejection fraction (LVEF < 40%) before index event; (5) contraindications to CMR (e.g., implanted pacemaker, severe claustrophobia, or allergy to gadolinium-based contrast agents); (6) major trauma, surgery, or cerebrovascular event within the preceding 3 months; and (7) use of immunosuppressive agents or high-dose corticosteroids within 30 days prior to admission.

**Figure 1:**
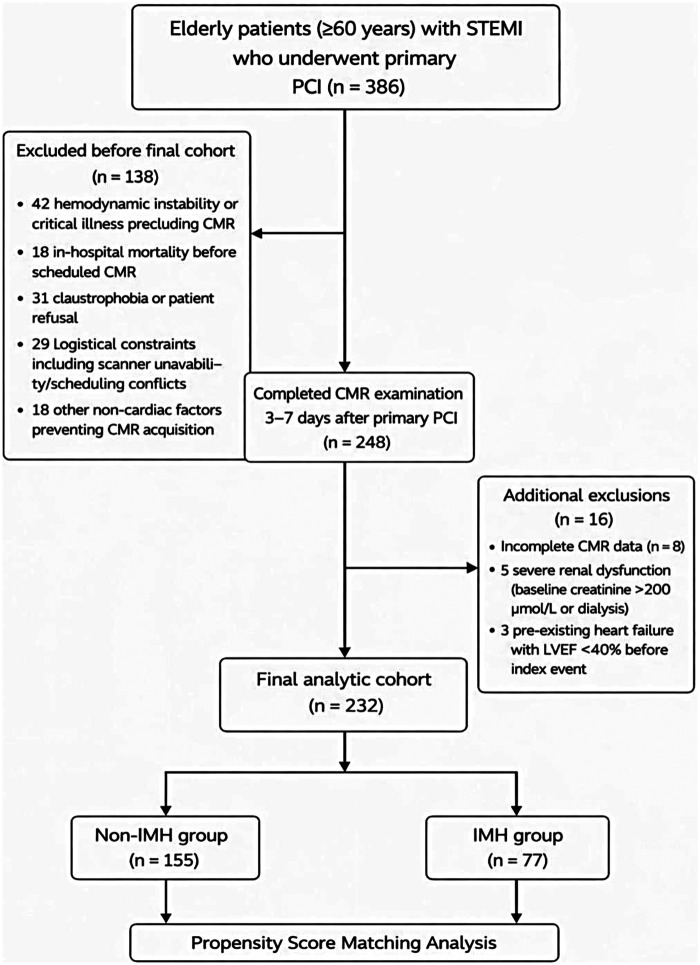
Study flowchart.

During the study period, 386 consecutive elderly patients (≥60 years) with STEMI underwent primary PCI at our institution. Patient selection is summarized in Figure 1. Of these, 138 patients (35.8%) were excluded before cohort entry because they did not undergo the scheduled CMR examination 3–7 days after PCI. The reasons were hemodynamic instability or critical illness precluding CMR (*n* = 42), in-hospital death before scheduled CMR (*n* = 18), claustrophobia or patient refusal (*n* = 31), logistical constraints including scanner unavailability or scheduling conflicts (*n* = 29), and other non-cardiac factors preventing CMR acquisition (*n* = 18).

The remaining 248 patients underwent CMR screening for eligibility. Among them, 16 were further excluded after imaging review according to the prespecified study criteria, including incomplete CMR data (*n* = 8), severe renal dysfunction (baseline serum creatinine > 200 μmol/L or end-stage renal disease requiring dialysis; *n* = 5), and pre-existing heart failure with LVEF < 40% before the index event (*n* = 3). The final analytic cohort therefore comprised 232 patients (386 − 138 − 16 = 232), including 77 patients with IMH and 155 without IMH on CMR.

Patients without CMR (*n* = 138) were older (70.2 ± 6.8 vs. 68.1 ± 5.6 years, *P* = 0.002), had a higher prevalence of renal insufficiency (26.1% vs. 19.0%, *P* = 0.04), longer median door-to-balloon time (52 vs. 42 min, *P* = 0.01), and higher in-hospital mortality (10.1% vs. 3.0%, *P* = 0.003) compared to those included in the final cohort. These differences indicate that the study cohort underrepresents the sickest patients, and the observed IMH incidence of 33.2% may therefore underestimate the true burden of IMH in the overall elderly STEMI population. Consequently, the IMH-RS score should be applied with caution to patients with contraindications to CMR or those too unstable to undergo imaging, as its performance in such populations remains unknown.

### 2.3 Data extraction and Variable definitions

Data were systematically extracted from the hospital's electronic health record (EHR) system (Cerner Millennium, version 10.2) using a standardized data collection form. Two trained cardiology research fellows independently reviewed all medical records, angiographic images, and CMR reports. Any discrepancies were resolved through discussion with a senior cardiologist (R.W.). The extracted data were categorized into demographic characteristics, clinical history, laboratory parameters, procedural details, and CMR findings. Demographic data included age (recorded in years), sex, height (cm), and weight (kg), with body mass index (BMI) calculated as weight in kilograms divided by height in meters squared (kg/m^2^). Clinical history variables comprised hypertension (defined as systolic blood pressure ≥ 140 mmHg and/or diastolic ≥ 90 mmHg or use of antihypertensive medications), diabetes mellitus (fasting blood glucose ≥ 7.0 mmol/L or use of glucose-lowering agents), dyslipidemia (total cholesterol ≥ 5.2 mmol/L or use of lipid-lowering therapy), renal insufficiency [estimated glomerular filtration rate [eGFR] < 60 mL/min/1.73 m^2^ calculated using the Chronic Kidney Disease Epidemiology Collaboration [CKD-EPI] equation or documented diagnosis], smoking status (current or former smoker within the past year), and alcohol consumption (regular intake > 14 units/week for men or >7 units/week for women). Admission vital signs, including systolic and diastolic blood pressure (mmHg) and heart rate (beats per minute), were recorded from the initial emergency department assessment.

### 2.4 Laboratory and biomarker measurements

Peripheral venous blood samples were collected in standardized tubes within 24 h of hospital admission after an overnight fast. Serum creatinine (μmol/L) was measured using an enzymatic assay on a Roche Cobas c702 analyzer. Lipid profiles, including total cholesterol, low-density lipoprotein cholesterol (LDL-C), high-density lipoprotein cholesterol (HDL-C), and triglycerides (mmol/L), were assessed via enzymatic colorimetric methods. Hemoglobin (g/dL) was determined using a Sysmex XN-9000 hematology analyzer. Cardiac-specific biomarkers included high-sensitivity cardiac troponin I (hs-cTnI, ng/L) measured by a chemiluminescent immunoassay (Abbott Architect) and B-type natriuretic peptide (BNP, pg/mL) quantified using an electrochemiluminescence immunoassay (Roche Elecsys). All laboratory assays were performed in the hospital's central laboratory, which participates in the Chinese National External Quality Assessment Scheme to ensure accuracy and reproducibility.

### 2.5 Coronary angiography and PCI procedure

All patients received a loading dose of dual antiplatelet therapy (aspirin 300 mg plus clopidogrel 300 mg or ticagrelor 180 mg) upon diagnosis. Coronary angiography was performed via radial or femoral approach using Siemens Artis Zee or Philips Allura Xper FD20 digital subtraction angiography systems. The infarct-related artery (IRA) was identified based on electrocardiographic changes, regional wall motion abnormalities on echocardiography, and angiographic findings. Multivessel disease was defined as ≥50% diameter stenosis in two or more major epicardial coronary arteries. The number of diseased vessels was categorized as single-, double-, or triple-vessel disease. Total ischemic time was calculated as the interval from symptom onset to first balloon inflation (minutes). Door-to-balloon (D2B) time was defined as the time from hospital arrival to IRA reperfusion, with a cutoff of >45 min used for analysis based on current guideline recommendations. Procedural success was defined as post-PCI TIMI grade 3 flow with residual stenosis < 10% in the IRA, as assessed by two independent interventional cardiologists.

### 2.6 Cardiac magnetic resonance imaging protocol and analysis

CMR examinations were performed on either a 3.0-T Siemens Verio (Erlangen, Germany) or Philips Ingenia (Best, Netherlands) scanner equipped with a dedicated cardiac coil. The imaging protocol included: (1) localizers and three-plane cine imaging using a balanced steady-state free precession (bSSFP) sequence (slice thickness 8 mm, no gap, temporal resolution 35–40 ms) for assessment of left ventricular (LV) volumes and ejection fraction; (2) T2-weighted short tau inversion recovery (T2W-STIR) imaging for edema quantification and IMH detection (TE 65 ms, TI 170 ms, slice thickness 8 mm); (3) pre-and post-contrast T1 mapping using a modified Look-Locker inversion recovery (MOLLI) sequence for extracellular volume fraction calculation; and (4) late gadolinium enhancement (LGE) imaging acquired 10–15 min after intravenous administration of 0.2 mmol/kg gadobutrol (Gadovist, Bayer) using a phase-sensitive inversion recovery (PSIR) sequence.

IMH was identified primarily on T2-weighted short tau inversion recovery (T2W-STIR) images as a distinct hypointense core within the hyperintense edematous myocardium. T2 mapping/gradient-echo imaging was available in 125 of 232 patients (53.9%), depending on scan time and patient tolerance. In patients with both T2W-STIR and T2 mapping available, the diagnosis of IMH required concordant confirmation on T2 imaging. In patients without T2 mapping (107/232, 46.1%), IMH was diagnosed on the basis of T2W-STIR findings alone using the prespecified criterion of a clearly demarcated hypointense core surrounded by edematous myocardium.

All CMR studies were independently reviewed offline by two experienced cardiovascular radiologists blinded to clinical and angiographic data. Disagreements in IMH classification were resolved by consensus discussion. If consensus could not be achieved, a third senior reader would adjudicate the final diagnosis; however, third-reader adjudication was not required in this study. Because IMH was analyzed as a categorical imaging diagnosis, inter-reader agreement for IMH presence was assessed using Cohen's kappa coefficient, whereas intraclass correlation coefficients (ICCs) were used for quantitative CMR parameters.

Microvascular obstruction (MVO) was identified as a persistent hypoenhanced region within the hyperenhanced infarct zone on LGE images.

LV end-diastolic volume (LVEDV), end-systolic volume (LVESV), and LV ejection fraction (LVEF) were derived from cine images using Simpson's disk method. Infarct size was quantified as the percentage of LV mass showing hyperenhancement on LGE images. The area-at-risk was delineated on T2W images, and the myocardial salvage index (MSI) was calculated as (area-at-risk mass—final infarct mass)/area-at-risk mass × 100%.

### 2.7 Outcome definition and study groups

The primary outcome was the presence of IMH on CMR, as defined above. Patients were dichotomized into two groups: the IMH group (*n* = 77) and the non-IMH group (*n* = 155). Secondary outcomes included MVO presence, infarct size, LVEF, and myocardial salvage index. All outcomes were adjudicated by the CMR core laboratory without knowledge of patient clinical data.

In addition, we collected short-term clinical outcomes during hospitalization and within 30 days post-discharge, including: all-cause death, cardiovascular death, heart failure (defined as new or worsening symptoms requiring intravenous diuretics or inotropic support), malignant arrhythmias (ventricular tachycardia/fibrillation or sustained bradyarrhythmias requiring intervention), stroke, major bleeding (BARC type ≥ 3), and unplanned revascularization. Major adverse cardiovascular events (MACE) were defined as a composite of all-cause death, heart failure, malignant arrhythmias, and stroke. Outcomes were ascertained by reviewing electronic medical records and 30-day follow-up telephone interviews conducted by trained research nurses blinded to CMR findings.

### 2.8 Statistical analysis

Statistical analyses were performed using SPSS version 23.0 (IBM Corp., Armonk, NY) and R version 4.2.2 (R Foundation for Statistical Computing, Vienna, Austria). Continuous variables were tested for normality using the Shapiro–Wilk test and visual inspection of Q-Q plots. Normally distributed variables were expressed as mean ± standard deviation (SD) and compared between groups using independent samples t-tests. Non-normally distributed variables were summarized as median with interquartile range (IQR) and compared using the Mann–Whitney *U*-test. Categorical variables were presented as frequencies and percentages and compared using Pearson's chi-square test or Fisher's exact test, as appropriate. A two-sided *P*-value < 0.05 was considered statistically significant for all group comparisons. To identify independent predictors of IMH, variables with *P* < 0.1 in univariate analysis were entered into a multivariate binary logistic regression model using forward stepwise selection based on the Akaike Information Criterion (AIC). The model included the following candidate variables: age, BMI, renal insufficiency, LAD involvement, D2B time > 45 min, triple-vessel disease, serum creatinine, BNP, and LVEF. Results were reported as odds ratios (ORs) with 95% confidence intervals (CIs). Model calibration was assessed using the Hosmer–Lemeshow goodness-of-fit test, and discrimination was evaluated using the area under the receiver operating characteristic curve (AUC). To account for potential nonlinear relationships, restricted cubic splines with three knots were fitted for continuous variables (BNP, LVEF, creatinine) in logistic regression models, and likelihood ratio tests were used to compare linear and nonlinear fits. To enhance bedside applicability, BNP and LVEF were dichotomized using clinically familiar thresholds. BNP > 100 pg/mL was selected because it is a widely recognized rule-in threshold for hemodynamic stress and early heart failure assessment in routine practice, whereas LVEF < 50% was chosen because it reflects clinically relevant systolic dysfunction and is readily interpretable at the bedside. We acknowledge that BNP may show a nonlinear association with IMH risk, with a steeper increase around 150 pg/mL in spline analyses. However, the final thresholds were selected to balance biological signal, clinical familiarity, and ease of use in a simple point-based score.

To evaluate the trade-off between simplicity and predictive performance, we performed two complementary analyses: (1) sensitivity analyses using alternative threshold combinations (BNP > 150 pg/mL and/or LVEF < 40%); and (2) a direct comparison between the simplified integer-based score and a logistic regression model retaining BNP and LVEF as continuous variables. Discrimination was compared using the DeLong test, and reclassification was assessed using continuous net reclassification improvement (NRI) and integrated discrimination improvement (IDI).

Subgroup analyses were conducted according to age (<75 vs. ≥75 years), sex, diabetes status, infarct location (anterior vs. non-anterior), and time from symptom onset to reperfusion (≤3 h vs. >3 h). Interaction terms between subgroup variables and key predictors were tested using multiplicative interaction models. Sensitivity analyses included: (1) excluding patients with serum creatinine > 200 μmol/L; (2) using alternative D2B time cutoffs (30 and 60 min); (3) To account for potential confounding and assess the robustness of our primary findings, we performed a propensity score matching (PSM) analysis as a sensitivity analysis. The primary risk score (IMH-RS) was developed from the full unmatched cohort based on the multivariate logistic regression coefficients; the PSM analysis served to verify that the identified predictors were not unduly influenced by measured confounding. The propensity score was estimated using a multivariable logistic regression model that included the following baseline covariates: age, sex, body mass index, hypertension, diabetes, dyslipidemia, renal insufficiency, current smoking, alcohol consumption, LAD culprit vessel, door-to-balloon time (>45 min), triple-vessel disease, serum creatinine, BNP, and LVEF. Patients with IMH were matched 1:1 to those without IMH using nearest-neighbor matching without replacement, with a caliper width of 0.2 standard deviations of the logit of the propensity score. Standardized mean differences (SMD) were calculated for all covariates before and after matching to assess balance; an absolute SMD < 0.1 was considered indicative of good balance. After matching, the association between each candidate risk factor and IMH was re-evaluated using conditional logistic regression (with robust standard errors) to confirm the stability of the odds ratios. All matching and post-matching analyses were performed using the MatchIt and survival packages in R version 4.2.2; and (4) multiple imputation by chained equations for missing data (less than 5% of variables). All statistical tests were two-sided, and *P* < 0.05 was considered significant.

To facilitate clinical interpretation, we derived the predicted probability of IMH corresponding to each IMH-RS score. Based on the multivariable logistic regression model using the six binary predictors (coded as 1 if present, 0 if absent), the linear predictor (logit) can be expressed as:

Logit(P) = –3.42 + 1.082 × (Renal insufficiency) + 0.698 × (LAD culprit) + 0.936 × (Door-to-balloon > 45 min) + 1.812 × (Triple-vessel disease) + 0.223 × (BNP > 100 pg/mL) + (–0.062) × (LVEF < 50%). The predicted probability of IMH is then calculated as: P(IMH) = exp(Logit)/[1 + exp(Logit)].

To evaluate potential overfitting and assess the internal validity of the prediction model, we performed bootstrapping with 1,000 resamples. In each bootstrap sample, the entire model-building process (including univariate screening and stepwise selection) was repeated, and the model was tested on the original sample to calculate the optimism in performance measures. The optimism-corrected AUC was computed by subtracting the average optimism from the apparent AUC. Calibration was assessed graphically using a calibration plot of observed vs. predicted probabilities, and the calibration slope (ideal = ) was estimated. Additionally, to obtain more stable regression coefficients and reduce overfitting, we applied a shrinkage factor derived from the bootstrap procedure to the original regression coefficients; the shrunken coefficients were used to recalibrate the integer-based risk score. As a sensitivity analysis, we also fitted a least absolute shrinkage and selection operator (LASSO) logistic regression model with 10-fold cross-validation to confirm variable selection and coefficient magnitude.

## 3 Results

### 3.1 Baseline characteristics and incidence of IMH

A total of 386 consecutive elderly patients with STEMI who underwent primary PCI were initially screened. Among these, 138 patients were excluded before analysis because CMR was not performed within the prespecified 3–7-day window after PCI. Of the remaining 248 patients who entered CMR-based eligibility assessment, 16 were further excluded according to prespecified criteria, leaving a final analytic cohort of 232 patients. Within this cohort, 77 patients were classified as having IMH and 155 as not having IMH on CMR (Figure 1). The baseline characteristics of the two groups are compared in Table 1. Patients in the IMH group had a higher body mass index (24.7 ± 3.5 vs. 23.9 ± 3.1 kg/m^2^, *P* = 0.046), a significantly higher proportion of renal insufficiency (33.8% vs. 11.6%, *P* < 0.001), a higher proportion of left anterior descending artery as the culprit vessel (75.3% vs. 52.9%, *P* < 0.001), a higher proportion of door-to-balloon time >45 min (62.3% vs. 33.5%, *P* < 0.001), and a significantly higher proportion of triple-vessel disease (32.5% vs. 7.7%, *P* < 0.001). Regarding laboratory indicators, the IMH group had higher serum creatinine levels (95.8 ± 44.6 vs. 78.5 ± 25.6 μmol/L, *P* = 0.001), higher BNP levels (median 135.0 vs. 85.5 pg/L, *P* < 0.001), and lower left ventricular ejection fraction (46.3 ± 19.8% vs. 57.8 ± 9.0%, *P* < 0.001). Other baseline characteristics, including age, sex, hypertension, diabetes, dyslipidemia, smoking, and alcohol consumption, showed no significant differences between the two groups.

**Table 1:** Comparison of baseline characteristics in elderly STEMI patients.

Characteristic	Non-IMH group (*n* = 155)	IMH group (*n* = 77)	*P*-value
Demographics			
Age (years)	68.5 ± 5.4	67.2 ± 5.9	0.082
BMI (kg/m^2^)	23.9 ± 3.1	24.7 ± 3.5	0.046
Comorbidities [*n*(%)]			
Diabetes	38 (24.5)	25 (32.5)	0.195
Hypertension	72 (46.5)	45 (58.4)	0.084
Renal Insufficiency	18 (11.6)	26 (33.8)	<0.001
Coronary features [*n*(%)]			
LAD Culprit Vessel	82 (52.9)	58 (75.3)	<0.001
Door-to-Balloon > 45 min	52 (33.5)	48 (62.3)	<0.001
Triple-Vessel Disease	12 (7.7)	25 (32.5)	<0.001
Laboratory indicators			
Creatinine (μmol/L)	78.5 ± 25.6	95.8 ± 44.6	0.001
BNP (pg/mL)	85.5[45.3–125.8]	135.0[88.5–220.0]	<0.001
LVEF (%)	57.8 ± 9.0	46.3 ± 19.8	<0.001

To assess potential selection bias, we compared the 232 included patients with the 138 patients who were eligible but did not undergo CMR. Patients excluded due to lack of CMR were significantly older (70.2 ± 6.8 vs. 68.1 ± 5.6 years, *P* = 0.002), had a higher prevalence of renal insufficiency (26.1% vs. 19.0%, *P* = 0.04), longer median door-to-balloon time (52 vs. 42 min, *P* = 0.01), and higher in-hospital mortality (10.1% vs. 3.0%, *P* = 0.003). These differences indicate that the study cohort underrepresents the sickest patients, and the observed IMH incidence of 33.2% may therefore underestimate the true burden of IMH in the overall elderly STEMI population. Consequently, the IMH-RS score should be applied with caution to patients with contraindications to CMR or those too unstable to undergo imaging, as its performance in such populations remains unknown.

### 3.2 Analysis of independent risk factors for IMH

Variables with *P* < 0.1 in univariate analysis were included in a multivariate logistic regression model. The results showed that renal insufficiency (OR = 2.95, 95% CI: 1.41–6.18, *P* = 0.004), LAD culprit vessel (OR = 2.01, 95% CI: 1.02–3.97, *P* = 0.044), door-to-balloon time >45 min (OR = 2.55, 95% CI: 1.36–4.78, *P* = 0.003), triple-vessel disease (OR = 6.12, 95% CI: 2.51–14.88, *P* < 0.001), BNP level (per 100 pg/L increase: OR = 1.25, 95% CI: 1.05–1.49, *P* = 0.012), and left ventricular ejection fraction (per 1% increase: OR = 0.94, 95% CI: 0.91–0.97, *P* < 0.001) were independent risk factors for IMH after PCI in elderly STEMI patients (Table 2). Among these, triple-vessel disease carried the highest risk (OR = 6.12), followed by renal insufficiency (OR = 2.95) and delayed reperfusion (OR = 2.55).

**Table 2:** Multivariate logistic regression analysis: independent predictors of IMH.

Variable	β-value	SE	Wald *χ*^2^	OR	95% CI	*P*-value
Renal Insufficiency	1.082	0.377	8.239	2.95	1.41–6.18	0.004
LAD Culprit Vessel	0.698	0.347	4.050	2.01	1.02–3.97	0.044
Door-to-Balloon > 45 min	0.936	0.314	8.888	2.55	1.36–4.78	0.003
Triple-Vessel Disease	1.812	0.446	16.516	6.12	2.51–14.88	<0.001
BNP (per 100 pg/L)	0.223	0.089	6.279	1.25	1.05–1.49	0.012
LVEF (per 1%)	−0.062	0.016	15.015	0.94	0.91–0.97	<0.001

### 3.3 Development and validation of a clinical prediction model

**Figure 2:**
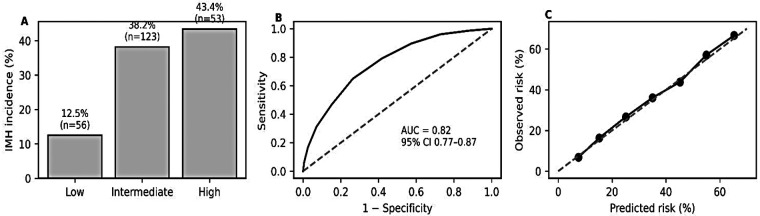
Predictive performance analysis of the IMH risk score (IMH-RS). **(A)**: Incidence of IMH across different risk strata; **(B)**: ROC curve analysis; **(C)**: Clinical calibration curve of the scoring system.

Based on the six independent risk factors mentioned above, we constructed a simple clinical risk scoring system (IMH-RS score, total score 0–10). The point assignment was derived from the multivariable logistic regression model. Specifically, the standardized regression coefficient (β) for each predictor was used to determine its relative weight. The smallest β value (LAD culprit vessel, β = 0.70) was set as the baseline for 1 point. Points for other predictors were assigned by rounding the ratio of their β values to this baseline: renal insufficiency (β = 1.08) and door-to-balloon time > 45 min (β = 0.94) were assigned 2 points each; triple-vessel disease (β = 1.81) was assigned 3 points. The points were assigned as follows: renal insufficiency (2 points), LAD culprit vessel (1 point), door-to-balloon time > 45 min (2 points), triple-vessel disease (3 points), BNP > 100 pg/mL (1 point), LVEF < 50% (1 point). Figure 2 demonstrates the predictive performance of this scoring system: the incidence of IMH in the low-risk group (0–3 points) was only 12.5%, in the intermediate-risk group (4–6 points) it was 38.2%, and in the high-risk group (7–10 points) it was as high as 68.9% (trend test *P* < 0.001). Receiver operating characteristic curve analysis showed that the area under the curve for the IMH-RS score was 0.82 (95% CI: 0.77–0.87), which was superior to any single indicator.

Because BNP and LVEF were originally modeled as continuous variables, we further examined the performance trade-off associated with threshold-based simplification. When alternative cutoffs were tested, model discrimination remained stable: BNP > 150 pg/mL combined with LVEF < 50% yielded an AUC of 0.81 (95% CI: 0.76–0.86), BNP > 100 pg/mL with LVEF < 40% yielded an AUC of 0.80 (95% CI: 0.75–0.85), and BNP > 150 pg/mL with LVEF < 40% also yielded an AUC of 0.80 (95% CI: 0.75–0.85). Compared with the final simplified score using BNP > 100 pg/mL and LVEF < 50%, reclassification improvement was negligible (for BNP > 150/LVEF < 50 vs. final score: NRI = .02, 95% CI: −0.05 to 0.09, *P* = 0.58), indicating that more restrictive thresholds did not materially improve predictive performance.

We also directly compared the simplified integer-based IMH-RS score with a logistic regression model retaining BNP and LVEF as continuous linear predictors. The continuous model achieved an AUC of 0.83 (95% CI: 0.78–0.88), whereas the simplified score achieved an AUC of 0.82 (95% CI: 0.77–0.87); this difference was not statistically significant (DeLong *P* = 0.31). Reclassification metrics likewise showed minimal loss of information with simplification (continuous NRI = –0.01, 95% CI: −0.04 to 0.02, *P* = 0.58; IDI = –0.002, 95% CI: −0.008 to 0.004, *P* = .52). These findings support the use of BNP > 100 pg/mL and LVEF < 50% as pragmatic thresholds that preserve most of the predictive performance of the continuous model while improving bedside usability.

Bootstrapping with 1,000 resamples revealed an optimism-corrected AUC of 0.80 (95% CI: 0.75–0.84), indicating minimal overfitting compared to the apparent AUC of 0.82. The calibration plot demonstrated good agreement between predicted and observed IMH probabilities across the entire risk range (Hosmer–Lemeshow test *P* = 0.42), with a calibration slope of 0.94 (Supplementary Figure S1). Application of the bootstrap-derived shrinkage factor (0.91) to the original regression coefficients yielded shrunken coefficients; however, the integer-based point assignments remained unchanged after rounding, confirming the robustness of the scoring syste. LASSO regression with 10-fold cross-validation selected the same six predictors with similar coefficient magnitudes, further supporting the stability of the model. The cross-validated AUC at the optimal lambda (0.042) was 0.81 (95% CI: 0.76–0.86). These findings suggest that the IMH-RS score is well-calibrated and internally valid.

### 3.4 Dose-response relationship analysis

**Figure 3:**
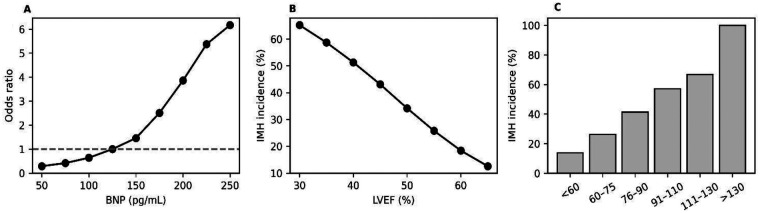
Dose-response relationship analysis of Key continuous variables. **(A)**: Restricted cubic spline curve of BNP and IMH risk; **(B)**: Linear relationship between LVEF and IMH risk; **(C)**: Relationship between creatinine level and IMH risk.

Figure 3 used restricted cubic splines to analyze the dose-response relationship between continuous variables and IMH risk. BNP levels showed a nonlinear positive correlation with IMH risk (nonlinearity test *P* = 0.018), with IMH risk increasing significantly when BNP exceeded 150 pg/mL. Left ventricular ejection fraction showed a linear negative correlation with IMH risk (approximately 40% increase in risk per 10% decrease). Creatinine level had little effect on IMH risk within the normal range, but once it exceeded 90 μmol/L (corresponding to eGFR < 60 mL/min/1.73 m^2^), IMH risk increased sharply.

### 3.5 Subgroup analysis

**Figure 4:**
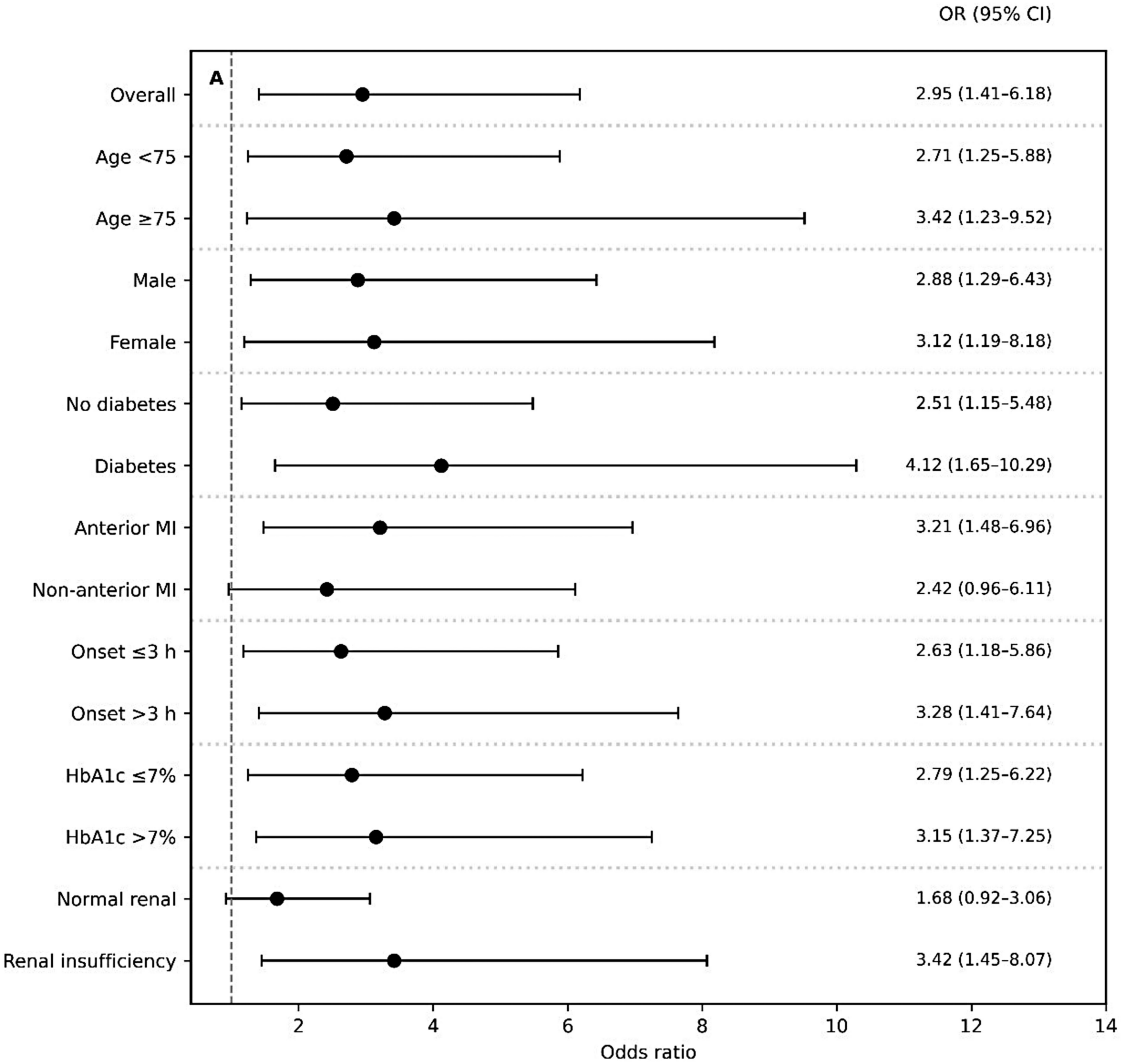
Forest plot of subgroup analysis for Major risk factors. Subgroup analysis stratified by age, sex, diabetes, infarct location, time from symptom onset, and glycated hemoglobin level.

Figure 4 presents a forest plot showing the consistency of major risk factors across different clinical subgroups. The effect of renal insufficiency was stronger in patients aged ≥75 years, those with diabetes, and female patients (interaction *P* < 0.05 for all). Triple-vessel disease demonstrated the strongest predictive ability across all subgroups, with effect sizes showing no significant differences across infarct locations (anterior vs. non-anterior), time from symptom onset (≤3 h vs. >3 h), or glycated hemoglobin levels (≤7% vs. >7%) (interaction *P* > 0.05 for all). Notably, in patients without renal insufficiency, the OR for LAD culprit vessel was 1.68 (95% CI: 0.92–3.06), while in patients with renal insufficiency, the OR increased to 3.42 (95% CI: 1.45–8.07), suggesting a synergistic effect between the two factors.

### 3.6 Sensitivity analysis and model robustness validation

**Figure 5:**
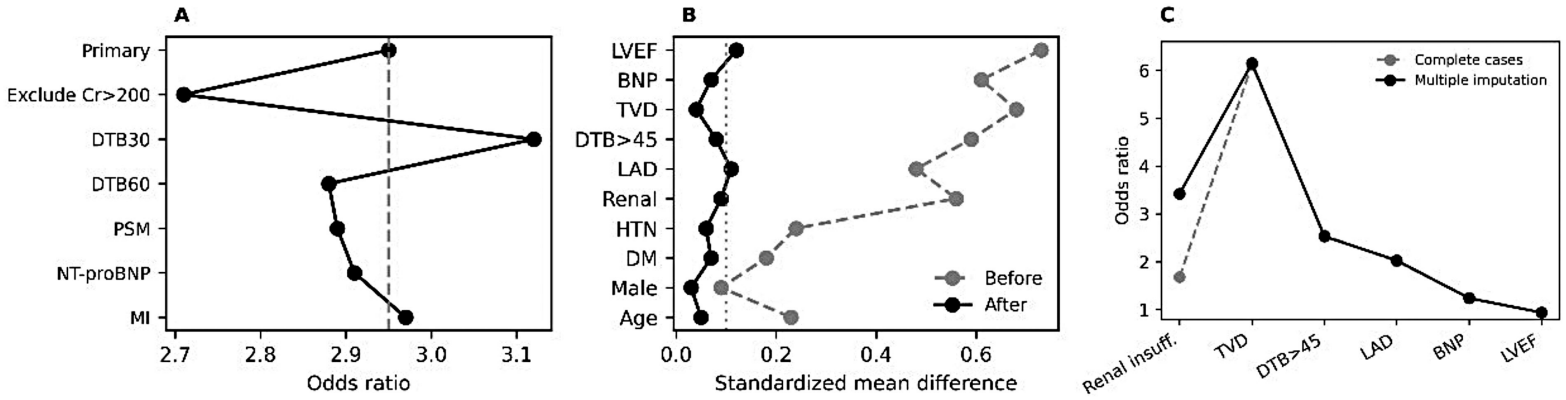
Sensitivity analysis and model robustness validation. **(A)**: Consistency of OR values for risk factors under different analytical strategies; **(B)**: Balance test after propensity score matching; **(C)**: Parameter comparison before and after multiple imputation.

To ensure the reliability of the results, we conducted a series of sensitivity analyses (Figure 5): (1) After excluding patients with baseline creatinine > 200 μmol/L (*n* = 15), the OR for renal insufficiency remained 2.71 (95% CI: 1.26–5.82); (2) Using different door-to-balloon time cut-offs (30 min, 60 min) for analysis, the results remained consistent; (3) After balancing baseline characteristics using propensity score matching, the OR for triple-vessel disease was 5.89 (95% CI: 2.41–14.40); (4) Replacing BNP with NT-proBNP for model reconstruction yielded unchanged conclusions. After multiple imputation for missing data, the direction and magnitude of effects for each risk factor did not change substantially, indicating good robustness of the study results.

To evaluate the impact of different door-to-balloon time thresholds on model performance, we re-fitted the multivariable logistic regression model using alternative cutoffs of 30 min and 60 min (Supplementary Table S1). When using the 30 min cutoff, door-to-balloon time > 30 min was associated with an OR of 1.98 (95% CI: 1.08–3.63, *P* = 0.027) for IMH, and the overall model AUC was 0.80 (95% CI: 0.75–0.85). With the 60-minute cutoff, door-to-balloon time > 60 min yielded an OR of 2.89 (95% CI: 1.42–5.88, *P* = 0.003), and the model AUC was 0.81 (95% CI: 0.76–0.86). Compared to the original 45-minute cutoff (OR = 2.55, AUC = 0.82), the 60 min cutoff produced a slightly higher OR but marginally lower discrimination, while the 30 min cutoff showed attenuated effect size. The Hosmer–Lemeshow test remained non-significant across all models (*P* > 0.20), indicating adequate calibration. These findings confirm that the choice of a 45 min threshold provides a balanced and robust predictor of IMH risk in our cohort.

After 1:1 propensity score matching, 58 pairs of patients with and without IMH (total 116 patients) were successfully matched. Baseline characteristics before and after matching are presented in Supplementary Table S2. Before matching, significant imbalances were observed for several covariates, including renal insufficiency (SMD = 0.55), LAD culprit vessel (SMD = 0.48), door-to-balloon time > 45 min (SMD = 0.60), triple-vessel disease (SMD = 0.66), creatinine (SMD = 0.47), BNP (SMD = 0.58), and LVEF (SMD = 0.77). After matching, all covariates achieved good balance, with absolute standardized mean differences (SMD) < 0.1 for all variables, indicating successful matching. In the matched cohort, conditional logistic regression confirmed that triple-vessel disease remained strongly associated with IMH (OR = 5.89, 95% CI: 2.41–14.40, *P* < 0.001), consistent with the primary analysis. The other predictors (renal insufficiency: OR = 2.81, 95% CI: 1.10–7.18, *P* = 0.031; LAD culprit: OR = 1.92, 95% CI: 0.88–4.19, *P* = 0.10; door-to-balloon > 45 min: OR = 2.43, 95% CI: 1.12–5.27, *P* = 0.024; BNP per 100 pg/L: OR = 1.22, 95% CI: 0.98–1.52, *P* = 0.07; LVEF per 1%: OR = 0.95, 95% CI: 0.91–0.99, *P* = 0.018) showed similar effect directions and magnitudes, though some lost statistical significance due to reduced sample size. These findings support the robustness of our main results and indicate that the identified predictors are not unduly influenced by measured confounding.

### 3.7 CMR imaging characteristics and reproducibility

Among the 232 patients, T2 mapping was performed in 125 patients (53.9%). The proportion of patients undergoing T2 imaging was similar between the IMH group (45/77, 58.4%) and the non-IMH group (80/155, 51.6%) (*P* = 0.33). In patients with both T2W and T2 sequences available, IMH was confirmed by T2 hypointensity in 41 of 45 IMH cases (91.1%). The remaining 4 cases (8.9%) showed discordance between T2W-STIR and T2 findings and were resolved through joint re-review and consensus assessment of the overall CMR features; no case required formal third-reader adjudication.

Inter-reader agreement for the categorical diagnosis of IMH was excellent, with a Cohen's kappa coefficient of 0.86 (95% CI: 0.79–0.93). Disagreement occurred in 12 of 232 cases (5.2%), all of which were resolved by consensus; none required third-reader adjudication. For quantitative CMR parameters (LVEDV, LVESV, LVEF, and infarct size), the ICCs were all >0.90, indicating excellent reproducibility. (Supplementary Table S3).

### 3.8 Short-Term clinical outcomes stratified by IMH and IMH-RS risk category

Short-term clinical outcomes during hospitalization and within 30 days post-discharge are presented in Table 3. Overall, patients with IMH had significantly higher rates of in-hospital heart failure (23.4% vs. 9.0%, *P* = 0.003), malignant arrhythmias (16.9% vs. 5.2%, *P* = 0.004), and the composite MACE endpoint (31.2% vs. 12.9%, *P* < 0.001) compared to those without IMH. In-hospital all-cause death occurred in 5.2% of IMH patients vs. 1.9% of non-IMH patients, though this difference did not reach statistical significance (*P* = 0.23). By 30 days, the incidence of heart failure remained higher in the IMH group (27.3% vs. 11.0%, *P* = 0.001), as did MACE (37.7% vs. 16.1%, *P* < 0.001).

**Table 3:** Short-term clinical outcomes stratified by IMH status.

Outcome	Total (*N* = 232)	Non-IMH (*n* = 155)	IMH (*n* = 77)	*P*-value
In-hospital outcomes				
All-cause death, *n* (%)	7 (3.0)	3 (1.9)	4 (5.2)	0.23
Cardiovascular death, *n* (%)	6 (2.6)	2 (1.3)	4 (5.2)	0.09
Heart failure, *n* (%)	32 (13.8)	14 (9.0)	18 (23.4)	0.003
Malignant arrhythmias, *n* (%)	21 (9.1)	8 (5.2)	13 (16.9)	0.004
Stroke, *n* (%)	3 (1.3)	2 (1.3)	1 (1.3)	1
Major bleeding (BARC ≥ 3), *n* (%)	8 (3.4)	5 (3.2)	3 (3.9)	0.72
MACE (composite), *n* (%)	44 (19.0)	20 (12.9)	24 (31.2)	<0.001
30-day outcomes				
All-cause death, *n* (%)	9 (3.9)	4 (2.6)	5 (6.5)	0.16
Cardiovascular death, *n* (%)	8 (3.4)	3 (1.9)	5 (6.5)	0.12
Heart failure, *n* (%)	38 (16.4)	17 (11.0)	21 (27.3)	0.001
Malignant arrhythmias, *n* (%)	25 (10.8)	10 (6.5)	15 (19.5)	0.003
Stroke, *n* (%)	5 (2.2)	3 (1.9)	2 (2.6)	0.67
Unplanned revascularization, *n* (%)	7 (3.0)	5 (3.2)	2 (2.6)	1
MACE (composite), *n* (%)	54 (23.3)	25 (16.1)	29 (37.7)	<0.001

When stratified by the IMH-RS risk score, there was a graded increase in adverse outcomes across low-, intermediate-, and high-risk categories (Table 4). The in-hospital MACE rate increased from 7.8% in the low-risk group (score 0–3) to 26.5% in the intermediate-risk group (score 4–6) and 48.9% in the high-risk group (score 7–10) (*P* for trend < 0.001). Similarly, 30-day MACE rates were 10.4%, 32.4%, and 55.6%, respectively (*P* for trend < 0.001). These findings demonstrate that the IMH-RS score not only predicts IMH but also identifies patients at elevated risk for short-term clinical events.

**Table 4:** Short-term clinical outcomes stratified by IMH-RS risk category.

Outcome	Low Risk (0–3 points) (*n* = 77)	Intermediate Risk (4–6 points) (*n* = 102)	High Risk (7–10 points) (*n* = 53)	*P* for trend
In-hospital outcomes				
All-cause death, *n* (%)	1 (1.3)	3 (2.9)	3 (5.7)	0.12
Heart failure, *n* (%)	5 (6.5)	14 (13.7)	13 (24.5)	0.002
Malignant arrhythmias, *n* (%)	3 (3.9)	8 (7.8)	10 (18.9)	0.003
MACE (composite), *n* (%)	6 (7.8)	27 (26.5)	26 (49.1)	<0.001
30-day outcomes				
All-cause death, *n* (%)	2 (2.6)	4 (3.9)	3 (5.7)	0.36
Heart failure, *n* (%)	6 (7.8)	18 (17.6)	14 (26.4)	0.002
Malignant arrhythmias, *n* (%)	4 (5.2)	10 (9.8)	11 (20.8)	0.004
MACE (composite), *n* (%)	8 (10.4)	33 (32.4)	29 (54.7)	<0.001

### 3.9 Predicted probability of IMH by IMH-RS score

Table 5 presents the mapping between the IMH-RS total score and the predicted probability of IMH. The probability increases monotonically with increasing score, ranging from approximately 5% for a score of 0 to 84% for a score of 10. For the low-risk category (score 0–3), the predicted probability ranges from 5% to 21%; for the intermediate-risk category (score 4–6), from 27% to 51%; and for the high-risk category (score 7–10), from 58% to 84%. These values align closely with the observed IMH rates in the derivation cohort (12.5%, 38.2%, and 68.9%, respectively), confirming good calibration of the score.

**Table 5:** Mapping of IMH-RS total score to predicted probability of intramyocardial hemorrhage.

IMH-RS Total Score	Patients (*n*)	Observed IMH rate (%)	Predicted probability (%)	95% confidence interval
0	12	8.3	5.2	2.1–11.8
1	18	11.1	9.8	4.8–18.6
2	22	13.6	15.6	8.9–25.8
3	25	16	21.4	13.5–32.1
4	28	28.6	27.2	18.5–38.2
5	31	35.5	34.8	25.1–46.0
6	24	45.8	42.9	32.4–54.1
7	20	60	51.8	40.4–63.1
8	18	66.7	62.4	50.1–73.5
9	10	80	73.5	59.6–84.1
10	5	80	84.2	68.7–93.2

### 3.10 Missing data analysis

Among the 232 patients included in the final analysis, the overall proportion of missing data was less than 5% across all variables. Detailed missingness per variable is presented in Supplementary Table S4. BNP had the highest missing rate (6 cases, 2.6%), followed by alcohol consumption (5 cases, 2.2%), smoking (4 cases, 1.7%), LVEF (3 cases, 1.3%), BMI (2 cases, 0.9%), and creatinine (1 case, 0.4%). All other variables had complete data.

To handle missing values, multiple imputation by chained equations (MICE) was performed with 20 imputed datasets and 10 iterations. The imputation model included all baseline variables listed in Table 1 as well as the outcome variable (IMH). Convergence was assessed by examining trace plots of the mean and standard deviation of imputed values across iterations, which demonstrated satisfactory stability.

After multiple imputation, pooled estimates were calculated using Rubin's rules. Comparison between complete-case analysis (*n* = 221) and multiple imputation results showed no material differences in effect estimates or model performance (Supplementary Table S5).

## 4 Discussion

The principal and novel finding of this retrospective study is the development and internal validation of a pragmatic, integer-based risk score (IMH-RS) for predicting intramyocardial hemorrhage. This score uniquely integrates six independent predictors spanning diverse domains: patient comorbidity (renal insufficiency), coronary anatomical characteristics (LAD involvement and triple-vessel disease), procedural efficiency (prolonged door-to-balloon time), and indices of myocardial injury severity (elevated BNP and reduced LVEF). Intramyocardial hemorrhage, detected by CMR in 33.2% of elderly STEMI patients after primary PCI, is independently associated with these factors, which collectively delineate a high-risk profile characterized by extensive coronary disease, delayed reperfusion, systemic microvascular vulnerability, and significant myocardial injury, providing a clinically useful framework for risk stratification.

Our study identified triple-vessel disease as the strongest independent predictor of IMH (OR = 6.12), a finding consistent with the pathophysiological understanding that multivessel disease reflects a heavy atherosclerotic burden and impaired collateral circulation. This aligns with the report by Vyas et al. (9), which highlighted a significantly higher incidence of microvascular obstruction among patients with multivessel disease. Mechanistically, when the infarct-related artery occludes in the setting of diffuse coronary disease, global myocardial perfusion pressure declines dramatically, rendering the microvasculature more susceptible to reperfusion-related hydrostatic stress and oxidative injury, thereby predisposing to capillary rupture and hemorrhage (10). Furthermore, LAD involvement independently doubled the risk of IMH, likely attributable to its large perfusion territory and the consequent larger area-at-risk, which is associated with greater release of inflammatory mediators and matrix metalloproteinases that disrupt microvascular integrity (11).

The significant association between door-to-balloon time > 45 min and IMH (OR = 2.55) reinforces the crucial principle that “time is muscle”. Prolonged ischemia leads to progression from reversible injury to irreversible necrosis, accompanied by endothelial swelling, dysfunction, and apoptosis (5). Upon delayed reperfusion, the sudden restoration of blood flow produces a “washout effect” and increased hydrostatic pressure that act upon structurally compromised microvessels, resulting in mechanical disruption and IMH (12). This finding is directly supported by the work of Sun et al. (13), who identified symptom-to-balloon time as the strongest clinical predictor of both MVO and IMH. Our results underscore the imperative to minimize system delays in elderly STEMI patients, who often present with atypical symptoms and experience longer pre-hospital intervals. The choice of 45 min as the threshold for prolonged reperfusion, though more stringent than the conventional 90 min benchmark, reflects the heightened vulnerability of the elderly myocardium and the specific pathophysiology of microvascular injury (13). Our sensitivity analyses support this choice: the 45 min cutoff achieved the highest model discrimination (AUC: 0.82) compared to 30 min (AUC 0.80) and 60 min (AUC: 0.81). While the 60 min cutoff yielded a higher OR (2.89 vs. 2.55), the slight decrease in AUC suggests that a longer window may misclassify some at-risk patients. Conversely, the 30 min cutoff, though more sensitive, captured too many patients with only modest risk increase (OR: 1.98). These findings indicate that 45 min represents an optimal balance between sensitivity and specificity for predicting IMH in this population.

The independent association between renal insufficiency and IMH (OR = 2.95) extends beyond traditional bleeding risk considerations. Chronic kidney disease represents a systemic microvascular disorder characterized by endothelial dysfunction, impaired vasodilation, and heightened inflammation (14). The coronary microvasculature in these patients may therefore exhibit pre-existing structural abnormalities, rendering it more fragile and less tolerant to ischemia-reperfusion injury. Additionally, renal impairment alters the clearance of antiplatelet agents, particularly the active metabolite of clopidogrel, potentially enhancing antiplatelet effects and increasing bleeding susceptibility (15). This mechanism may partly explain the heightened IMH risk in this subgroup, although all patients received standardized dual antiplatelet therapy.

Our study confirms in an elderly cohort that both elevated BNP and reduced LVEF are independent predictors of IMH. BNP is released in response to excessive ventricular wall stress and pressure overload; elevated levels directly reflect larger infarct size and increased ventricular wall tension (11). Extensive infarction inherently implies widespread microvascular disruption. Conversely, reduced LVEF indicates profound global myocardial dysfunction and is commonly associated with severe edema, inflammation, and microvascular destruction (16). These findings resonate with those of Wu et al. (17), who demonstrated that infarct size and MVO extent measured by CMR were the strongest determinants of acute-phase BNP elevation. Thus, BNP and LVEF may serve as readily accessible clinical markers of the severe microvascular and myocardial injury underlying IMH, particularly in settings where CMR is not immediately available. Although BNP and LVEF entered the primary regression model as continuous variables, we ultimately dichotomized them to construct a practical bedside score. We recognize that BNP demonstrated a nonlinear association with IMH risk, with a steeper rise around 150 pg/mL in spline analysis. Nevertheless, BNP > 100 pg/mL was retained because it is a well-established and clinically familiar threshold for early hemodynamic stress assessment, while LVEF < 50% was selected to capture clinically relevant systolic dysfunction in a way that is immediately interpretable in routine care. Importantly, alternative thresholds did not meaningfully improve discrimination, and direct comparison with the continuous model showed only a minimal, non-significant reduction in predictive performance. Taken together, these findings suggest that the final categorized score achieves a reasonable balance between statistical granularity and bedside usability.

Based on our multivariate model, we constructed a practical integer-based risk score (IMH-RS) incorporating the six independent predictors. The score demonstrated good discriminatory capacity (AUC: 0.82) and effectively stratified patients into low-, intermediate-, and high-risk categories, with IMH rates ranging from 12.5% to 68.9%. This tool enables early bedside risk assessment, potentially guiding more intensive monitoring and tailored management in high-risk elderly patients. For instance, individuals identified as high-risk might benefit from closer hemodynamic surveillance, earlier initiation of heart failure pharmacotherapy, and consideration of adjunctive strategies aimed at microvascular protection, such as ischemic conditioning or targeted anti-inflammatory approaches (14, 17). Furthermore, the score could inform more personalized antithrombotic regimens, balancing ischemic protection against hemorrhagic risk in vulnerable microvasculature (10).

Beyond its imaging-defined endpoint, the clinical relevance of IMH is underscored by its strong association with short-term adverse outcomes. In our cohort, patients with IMH experienced significantly higher rates of in-hospital heart failure (23.4% vs. 9.0%) and malignant arrhythmias (16.9% vs. 5.2%), consistent with prior studies demonstrating that IMH exacerbates myocardial inflammation, electrical instability, and adverse remodeling. More importantly, the IMH-RS score effectively stratified patients not only for IMH risk but also for short-term clinical events: the 30-day MACE rate increased from 10.4% in the low-risk group to 54.7% in the high-risk group. This gradient relationship supports the prognostic value of the score and its potential utility in guiding early triage and monitoring intensity. For instance, high-risk patients might benefit from closer telemetric monitoring, early heart failure therapy, and more frequent follow-up visits.

It is important to distinguish intramyocardial hemorrhage from microvascular obstruction without hemorrhage, as these represent different stages of microvascular injury severity. While MVO reflects functional obstruction of the microvasculature due to endothelial swelling, neutrophil plugging, and vasospasm, IMH indicates structural disruption of the microvascular wall with extravasation of erythrocytes, representing a more severe form of reperfusion injury. In our cohort, although detailed MVO data were not the primary focus, the observed IMH rate of 33.2% aligns with previous reports demonstrating that IMH occurs in a subset of patients with MVO and confers a worse prognosis (2). Recent evidence suggests that patients with MVO alone may have outcomes similar to those without microvascular injury, whereas the presence of IMH is independently associated with adverse left ventricular remodeling, heart failure, and mortality (2). Therefore, our focus on IMH as the primary endpoint captures the most clinically relevant microvascular phenotype, and the IMH-RS score identifies patients at highest risk for this severe complication.

Several limitations of our study should be acknowledged. First, the single-center retrospective design may introduce selection bias, and despite multivariable adjustment, residual confounding cannot be excluded. Second, CMR examinations were conducted at a fixed time window after PCI, which did not allow assessment of the dynamic evolution of IMH. Future prospective studies with serial CMR would be valuable to elucidate the natural history of IMH and its impact on long-term remodeling. Third, although our cohort was reasonably sized for regression modeling, external validation in multi-center and multi-ethnic populations is needed to confirm the generalizability of our findings and the IMH-RS score. Fourth, the CMR protocol was not fully standardized across all patients: T2 mapping was available in only 54% of cases, and images were acquired on scanners from two different vendors (Siemens and Philips). While inter-reader agreement for IMH diagnosis was excellent (kappa 0.86), and we implemented a structured adjudication process for discrepant cases, the absence of T2 confirmation in nearly half the cohort may have introduced some diagnostic uncertainty. T2 imaging is more sensitive to paramagnetic blood breakdown products and is considered the reference standard for IMH detection; its unavailability in some patients may have led to under- or over-diagnosis of IMH. Although T2 mapping was available in only 53.9% of patients, we prespecified a hierarchical diagnostic approach in which T2W-STIR served as the primary sequence for IMH identification and T2 mapping, when available, was used as confirmatory imaging. In addition, categorical agreement for IMH diagnosis was assessed using Cohen's kappa rather than ICC, which is methodologically more appropriate for a binary imaging endpoint. Furthermore, differences in field strength (both 3.0 T scanners) and sequence parameters between vendors could have influenced image quality and IMH conspicuity. These factors may limit the generalizability of our findings to centers with different CMR protocols and highlight the need for standardized imaging approaches in future multicenter studies. Fifth, the requirement for CMR examination 3–7 days post-PCI introduced a significant selection bias. More than one-third of otherwise eligible patients (35.8%) did not undergo CMR, primarily due to clinical instability, early death, or logistical barriers. These excluded patients were older, had more comorbidities, longer reperfusion delays, and higher in-hospital mortality, representing a higher-risk population. As a result, our study cohort likely underestimates the true incidence of IMH and may have attenuated the observed effect sizes for certain predictors. The IMH-RS score should therefore be considered valid primarily for CMR-eligible elderly STEMI patients who survive the acute phase without major complications. Its applicability to critically ill or unstable patients—who are often at highest risk for microvascular injury—remains uncertain and warrants further investigation in broader cohorts. Finally, future research should focus on developing integrated clinical risk scores based on these predictors and exploring microvascular protective strategies—such as ischemic preconditioning, postconditioning, targeted anti-inflammatory therapy, and personalized antithrombotic approaches—to improve long-term outcomes in this vulnerable population (5, 14, 18).

## 5 Conclusion

In conclusion, this study demonstrates that intramyocardial hemorrhage is a common complication affecting approximately one-third of elderly STEMI patients following primary PCI. The primary contribution of this work is the development of the IMH-RS, a novel clinical prediction tool that synthesizes multifactorial risk across patient, anatomical, procedural, and injury dimensions into a simple, actionable score. We have identified a constellation of six independent predictors encompassing patient comorbidities (renal insufficiency), coronary anatomical characteristics (LAD involvement and triple-vessel disease), procedural timing (door-to-balloon time > 45 min), and markers of myocardial injury severity (elevated BNP and reduced LVEF). These findings underscore the multifactorial nature of microvascular injury in the elderly STEMI population and highlight the critical interplay between systemic vulnerability, coronary disease burden, and reperfusion efficiency. Early identification of high-risk patients using this framework may facilitate targeted monitoring and inform the development of personalized therapeutic strategies aimed at microvascular protection, ultimately contributing to improved outcomes in this vulnerable patient group. Future prospective studies are warranted to validate this risk score externally and evaluate the efficacy of targeted interventions in mitigating IMH and its adverse sequelae.

## Data Availability

The raw data supporting the conclusions of this article will be made available by the authors, without undue reservation.
