# Supplementary material for: Development of a risk score for intramyocardial hemorrhage in elderly STEMI patients after primary PCI: a retrospective cohort study with propensity score matching analysis
Source: Front Cardiovasc Med. 2026 Jul 3;13:1786172. doi: 10.3389/fcvm.2026.1786172 (PMC13375873; doi:10.3389/fcvm.2026.1786172)
Supplement: Supplementary file 1 [file Supplementaryfile1.docx]

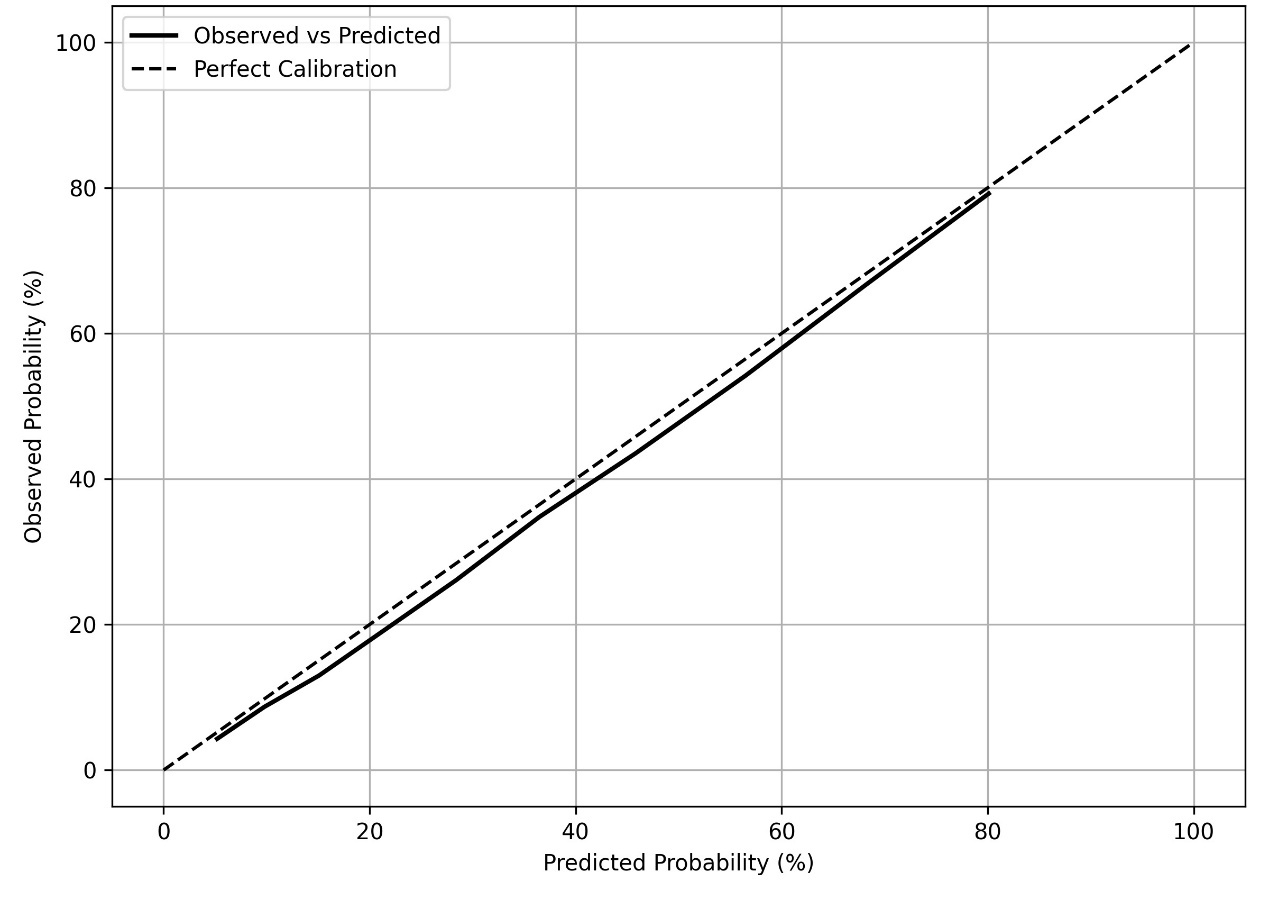


**Supplementary Figure S1. Calibration plot of the IMH-RS score for predicting intramyocardial hemorrhage.**
