# Supplementary material for: Development of a risk score for intramyocardial hemorrhage in elderly STEMI patients after primary PCI: a retrospective cohort study with propensity score matching analysis
Source: Front Cardiovasc Med. 2026 Jul 3;13:1786172. doi: 10.3389/fcvm.2026.1786172 (PMC13375873; doi:10.3389/fcvm.2026.1786172)
Supplement: Supplementary file 2 [file Table1.docx]

**Supple Table S1. Sensitivity Analysis for Door-to-Balloon Time Cutoffs**

| **D2B Cutoff** | **OR (95% CI)** | **P-value** | **Model AUC (95% CI)** | **Hosmer–Lemeshow P** |
| --- | --- | --- | --- | --- |
| >30 minutes | 1.98 (1.08–3.63) | 0.027 | 0.80 (0.75–0.85) | 0.31 |
| >45 minutes* | 2.55 (1.36–4.78) | 0.003 | 0.82 (0.77–0.87) | 0.42 |
| >60 minutes | 2.89 (1.42–5.88) | 0.003 | 0.81 (0.76–0.86) | 0.28 |

**Supplementary Table S2. Variables Included in Propensity Score Estimation and Covariate Balance Before and After Matching**

| **Variable** | **Before Matching** |  |  | **After Matching** |  |  |
| --- | --- | --- | --- | --- | --- | --- |
|  | IMH (n=77) | Non-IMH (n=155) | SMD | IMH (n=58) | Non-IMH (n=58) | SMD |
| Age, years | 67.2 ± 5.9 | 68.5 ± 5.4 | 0.23 | 67.8 ± 5.6 | 68.0 ± 5.5 | 0.04 |
| Male, n (%) | 50 (64.9) | 102 (65.8) | 0.02 | 38 (65.5) | 39 (67.2) | 0.04 |
| BMI, kg/m² | 24.7 ± 3.5 | 23.9 ± 3.1 | 0.24 | 24.3 ± 3.3 | 24.2 ± 3.2 | 0.03 |
| Hypertension, n (%) | 45 (58.4) | 72 (46.5) | 0.24 | 30 (51.7) | 29 (50.0) | 0.03 |
| Diabetes, n (%) | 25 (32.5) | 38 (24.5) | 0.18 | 16 (27.6) | 15 (25.9) | 0.04 |
| Dyslipidemia, n (%) | 30 (39.0) | 55 (35.5) | 0.07 | 21 (36.2) | 20 (34.5) | 0.04 |
| Renal insufficiency, n (%) | 26 (33.8) | 18 (11.6) | 0.55 | 12 (20.7) | 11 (19.0) | 0.04 |
| Current smoking, n (%) | 42 (54.5) | 80 (51.6) | 0.06 | 31 (53.4) | 30 (51.7) | 0.03 |
| Alcohol consumption, n (%) | 18 (23.4) | 35 (22.6) | 0.02 | 13 (22.4) | 12 (20.7) | 0.04 |
| LAD culprit vessel, n (%) | 58 (75.3) | 82 (52.9) | 0.48 | 37 (63.8) | 36 (62.1) | 0.04 |
| Door-to-balloon >45 min, n (%) | 48 (62.3) | 52 (33.5) | 0.6 | 28 (48.3) | 27 (46.6) | 0.03 |
| Triple-vessel disease, n (%) | 25 (32.5) | 12 (7.7) | 0.66 | 11 (19.0) | 10 (17.2) | 0.05 |
| Creatinine, μmol/L | 95.8 ± 44.6 | 78.5 ± 25.6 | 0.47 | 86.2 ± 30.1 | 85.4 ± 28.9 | 0.03 |
| BNP, pg/L | 135.0 [88.5–220.0] | 85.5 [45.3–125.8] | 0.58 | 112.0 [70.0–168.0] | 108.0 [68.0–160.0] | 0.04 |
| LVEF, % | 46.3 ± 19.8 | 57.8 ± 9.0 | 0.77 | 52.5 ± 14.2 | 53.1 ± 13.8 | 0.04 |

**Supplementary Table S3.** Inter-reader reproducibility for CMR parameters.

| Parameter | ICC (95% CI) | Kappa (95% CI) |
| --- | --- | --- |
| Categorical Diagnosis |  |  |
| IMH presence | – | 0.86 (0.79–0.93) |
| MVO presence | – | 0.84 (0.76–0.91) |
| Quantitative Measures |  |  |
| LVEDV | 0.94 (0.91–0.96) | – |
| LVESV | 0.93 (0.90–0.95) | – |
| LVEF | 0.95 (0.93–0.97) | – |
| Infarct size (%LV) | 0.91 (0.87–0.94) | – |
| Area-at-risk (%LV) | 0.89 (0.84–0.93) | – |

**Supplementary Table S4. Missing Data by Variable**

| **Variable** | **Total N** | **Missing (n)** | **Missing (%)** | **Variable Type** | **Imputation Method** |
| --- | --- | --- | --- | --- | --- |
| Age | 232 | 0 | 0 | Continuous | Not imputed |
| Sex | 232 | 0 | 0 | Binary | Not imputed |
| BMI | 232 | 2 | 0.9 | Continuous | Predictive mean matching |
| Hypertension | 232 | 0 | 0 | Binary | Not imputed |
| Diabetes | 232 | 0 | 0 | Binary | Not imputed |
| Dyslipidemia | 232 | 3 | 1.3 | Binary | Logistic regression |
| Renal insufficiency | 232 | 0 | 0 | Binary | Not imputed |
| Smoking | 232 | 4 | 1.7 | Binary | Logistic regression |
| Alcohol consumption | 232 | 5 | 2.2 | Binary | Logistic regression |
| LAD culprit vessel | 232 | 0 | 0 | Binary | Not imputed |
| Door-to-balloon time | 232 | 0 | 0 | Continuous | Not imputed |
| Triple-vessel disease | 232 | 0 | 0 | Binary | Not imputed |
| Creatinine | 232 | 1 | 0.4 | Continuous | Predictive mean matching |
| BNP | 232 | 6 | 2.6 | Continuous | Predictive mean matching (log-transformed) |
| LVEF | 232 | 3 | 1.3 | Continuous | Predictive mean matching |
| IMH (outcome) | 232 | 0 | 0 | Binary | Not imputed |

**Supplementary Table S5. Comparison of Model Performance and Effect Estimates Before and After Multiple Imputation**

| **Metric** | **Complete-Case Analysis (n=221)** | **Multiple Imputation (n=232)** | **Difference** |
| --- | --- | --- | --- |
| AUC (95% CI) | 0.82 (0.77–0.87) | 0.82 (0.77–0.87) | 0 |
| Renal insufficiency OR (95% CI) | 2.91 (1.38–6.12) | 2.95 (1.41–6.18) | 0.04 |
| LAD culprit vessel OR | 1.98 (1.00–3.92) | 2.01 (1.02–3.97) | 0.03 |
| Door-to-balloon >45 min OR | 2.52 (1.34–4.74) | 2.55 (1.36–4.78) | 0.03 |
| Triple-vessel disease OR | 6.08 (2.48–14.82) | 6.12 (2.51–14.88) | 0.04 |
| BNP (per 100 pg/L) OR | 1.24 (1.04–1.48) | 1.25 (1.05–1.49) | 0.01 |
| LVEF (per 1%) OR | 0.94 (0.91–0.97) | 0.94 (0.91–0.97) | 0 |
| Hosmer–Lemeshow P-value | 0.45 | 0.42 | –0.03 |
